# Predominance of *Cronobacter sakazakii* Sequence Type 4 in Neonatal Infections

**DOI:** 10.3201/eid1709.110260

**Published:** 2011-09

**Authors:** Susan Joseph, Stephen J. Forsythe

**Affiliations:** Author affiliation: Nottingham Trent University, Nottingham, UK

**Keywords:** bacteria, Cronobacter sakazakii, bacteria, multilocus sequence typing (MLST), neonatal meningitis, United Kingdom, dispatch

## Abstract

A 7-loci (3,036 nt) multilocus sequence typing scheme was applied to 41 clinical isolates of *Cronobacter sakazakii*. Half (20/41) of the *C. sakazakii* strains were sequence type (ST) 4, and 9/12 meningitis isolates were ST4. *C. sakazakii* ST4 appears to be a highly stable clone with a high propensity for neonatal meningitis.

*Cronobacter* is a genus within the family *Enterobacteriaceae* and was previously known as *Enterobacter sakazakii*. It is closely related to the genera *Enterobacter* and *Citrobacter*. *Cronobacter* spp. have been frequently isolated from the environment, plant material (wheat, rice, herbs, and spices), and various food products, including powdered infant formula (PIF). *Cronobacter* spp. have come to prominence because of their association with severe neonatal infections, which can be fatal (*1**–**3*). Our current knowledge of the virulence and epidemiology of this organism is limited. However, because neonates are frequently fed reconstituted PIF, this product has been the focus of attention for reducing infection risk to neonates because the number of exposure routes is limited (*1**,**2*).

Infections with *Cronobacter* spp. occur across all age groups, and most infections, albeit less severe, are in the adult population. However, neonates, particularly those of low birthweight, are the major identified group at risk, because the organism can cause meningitis, necrotizing enterocolitis (NEC), and sepsis in patients in neonatal intensive care units and has a high mortality rate (*1**–**3*). Bowen and Braden (*4*) reviewed 46 cases of invasive (non-NEC) infant *Cronobacter* infections to define risk factors and provide guidance for prevention and treatment. Although these infections have been associated with intrinsically and extrinsically contaminated PIF, other environmental sources are possible and several non–infant formula–associated cases have been reported (*5*). *Cronobacter* spp. have been shown to invade human intestinal cells, replicate in macrophages, and invade the blood–brain barrier (*6*). Kucerova et al. (*7**,**8*) used comparative genomic hybridization-based analysis to describe a range of virulence traits in *Cronobacter* spp., including iron acquisition mechanisms, fimbriae, and macrophage survival.

Recently, Baldwin et al. (*9*) constructed a comprehensive multilocus sequence typing (MLST) scheme for *Cronobacter* spp. based on 7 housekeeping genes (*atpD, fusA, glnS, gltB, gyrB, infB, ppsA*; 3,036 nt concatenated length). The MLST scheme currently has 66 defined sequence types covering all *Cronobacter* spp. (www.pubMLST.org/cronobacter). However, the scheme has not been applied for any epidemiologic purposes. Therefore, we investigated whether severity of infection by *Cronobacter* spp. is associated with particular genotype(s) by compiling patient details, isolation site, and clinical signs for clinical *C. sakazakii* isolates and comparing these with the sequence type (ST) profile of the isolates.

## The Study

**Error! Hyperlink reference not valid.**Forty-one clinical *C. sakazakii* strains were included in the study. These strains were from 7 countries and had been isolated during 1953–2008. The strains included those of recent (*1**–**3**,**10**–**12*) and those of more historic interest (>25 years 13–15; ). Strains used in this study, along with patient details and clinical signs, are shown in Table 1. Details of clinical signs were collated from information in the associated publication or supplied by the strain provider (Centers for Disease Control and Prevention, Atlanta, GA, USA). Primers and conditions for amplification and sequencing of the 7 MLST genes *atpD* (390 bp), *fusA* (438 bp), *glnS* (363 bp), *gltB* (507 bp), *gyrB* (402 bp), *infB* (441 bp) and *ppsA* (495 bp) were as described (*9*). All sequences are available for download and independent analysis through open access at www.pubMLST.org/cronobacter.

**Table 1:** Strains used in study of *Cronobacter sakazakii* genotypes and disease severity and clinical details derived from original case histories*

Strain	Patient type/age (EGA)†	Clinical signs/outcome	Isolation site	Year	Country	ST	Reference
553	Neonate/1 d	UNK	UNK	1977	Netherlands	4	(*15*)
557	Neonate/5 d	UNK	UNK	1979	Netherlands	4	(*15*)
693	Neonate/13 d (41 wk)	Asymptomatic	Feces	1994	France	13	(*3*)
695	Neonate/15 d (32 wk)	Fatal NEC II	Trachea	1994	France	4	(*3**,**6*)
701	Neonate/28 d (28 wk)	Fatal NEC III	Peritoneal fluid	1994	France	4	(*3**,**6*)
709	Neonate/18 d (29 wk)	Septicemia	Trachea	1994	France	4	(*3**,**6*)
767	Neonate/19 d (31 wk)	Fatal meningitis	Trachea	1994	France	4	(*3**,**6*)
721	Neonate/2 wk	Meningitis	CSF	2003	USA	4	
978	Neonate/<1 wk	UNK	Enteral feeding tube	2007	UK	3	(*12*)
696	Neonate/17 d (32 wk)	NEC II	Feces	1994	France	12	(*3**,**6*)
984	Neonate/3–4 wk	UNK	Enteral feeding tube	2007	UK	3	(*12*)
690	Neonate/27 d (31 wk)	Asymptomatic	Feces	1994	France	12	(*3*)
1218	Neonate/<1 mo (30 wk)	Fatal meningitis	CSF	2001	USA	1	
1219	Neonate/<1 mo (36 wk)	Fatal meningitis	CSF	2002	USA	4	
1221	Neonate/<1 mo	Meningitis, adverse neurologic outcome	CSF	2003	USA	4	
1225	Neonate/<1 mo (35 wk)	Fatal meningitis	Blood	2007	USA	4	
1231	Neonate (33 wk)	Fatal neurologic damage	Feces	2004	New Zealand	4	(*2*)
HPB 3290	Neonate (33 wk)	Meningitis	CSF	2001	USA	1	(*1*)
1249	Neonate	Fatal infection	UNK	2009	UK	31	
1220	Infant/6 wk (37 wk)	Brain abscess, nonfatal	CSF	2003	USA	4	
1223	Infant/6 wk (31 wk)	UNK, in ICU	Blood	2004	USA	4	
1240	Infant/7 wk	Fatal meningitis	CSF	2008	USA	4	(*11*)
1242	Infant/7 wk	Fatal meningitis	Brain	2008	USA	4	(*11*)
1241	Infant/7 mo	Sudden infant death syndrome	Blood	2008	USA	1	(*11*)
1222	Infant/8 mo	Fever, recovered	Blood	2003	USA	4	
1224	Infant/10 mo	Fever, severe combined immunodeficiency	Blood	2004	USA	4	
HPB 2856	Child/6 y	UNK	UNK	2002	Canada	15	(*10*)
ATCC 29544	Child	UNK	Throat	1980	USA	8	(*13*)
20	Child/6 y	UNK	Feces	2004	Czech Rep	4	
12	Adult/74 y	UNK	Feces	2004	Czech Rep	1	
CDC 0743–75	UNK	Foot wound	Wound	1975	USA	41	(*13*)
CDC 407–77	UNK	UNK	Sputum	1977	USA	8	(*13*)
CDC 996–77	UNK	UNK	Spinal fluid	1977	USA	8	(*13*)
NCTC 9238	UNK	UNK	Abdomen pus	1953	UK	18	(*15*)
HPB 2852	UNK	UNK	UNK	1990	Canada	8	(*10*)
HPB 2853	UNK	UNK	UNK	1990	Canada	4	(*10*)
511	UNK	UNK	UNK	1983	Czech Rep	8	(*14*)
513	UNK	UNK	UNK	1983	Czech Rep	8	(*14*)
520	UNK	UNK	UNK	1983	Czech Rep	12	(*14*)
526	UNK	UNK	UNK	1983	Czech Rep	8	(*14*)
558	UNK	UNK	UNK	1983	Netherlands	4	(*15*)

*EGA, estimated gestational age; ST, sequence type; UNK, unknown; NEC, necrotizing enterocolitis; CSF, cerebrospinal fluid; ICU, intensive care unit; Czech Rep, Czech Republic; CDC, Centers for Disease Control and Prevention. †Values <37 weeks are considered premature.

Comparative analysis with the online *Cronobacter* MLST database (covering isolates from all sources) showed that the clinical isolates were in 10 of 30 STs defined for *C. sakazakii* spp. However, the clinical strains were not evenly distributed across the STs. Of particular interest was that half (20/41) of the strains were ST4 (Table 2). The remaining strains were ST8 (7), ST1 (4), ST12 (3), ST3 (2), ST13, ST15, ST18, ST31, and ST41 (1 each). Of the 20 ST4 strains, 10 were from neonates, 7 from infants, and 1 from a child; 2 had no patient details. Similarly, most (9/12) isolates from meningitis cases were ST4 strains; 7 were isolated from cerebrospinal fluid and the others from blood and the trachea. The remaining ST4 strains were from bacteremia cases (1), NEC (2), and undefined infection (1), with 6 from unknown sources. ST4 was the main ST associated with neonates (10/18); this ST has been reported by Baldwin et al. (*9*) for the high incidence of PIF isolates.

**Table 2:** Summary of *Cronobacter sakazakii* sequence types and source details from study of *Cronobacter sakazakii* genotypes and disease severity*

ST	No. infections	Patient details						Clinical signs					
		Neonate†	Infant‡	Child	Adult	UNK		Meningitis	Bacteremia	NEC	Infection	Asymptomatic	UNK
1	4	2	1		1			2	1				1
3	2	2											2
4	20	10	7	1		2		9	1	2	2		6
8	7			1		6		1					6
12	3	2				1				1		1	1
13	1	1										1	
15	1			1									1
18	1					1					1		
31	1	1									1		
41	1					1					1		
Total	41	18	8	3	1	11		12	2	3	5	2	17

*ST, sequence type; UNK, unknown; NEC, necrotizing enterocolitis. †Age <28 d. ‡Age 28–364 d.

The ST4 clinical strains were from 6 countries (the Netherlands, France, United States, New Zealand, Czech Republic, and Canada) and had been isolated during 1977–2008 (Table 1). Of the 30 strains with known patient details, only 1 isolate (ST1) was from an adult patient. To date, all other isolates from adults have been identified as *C. malonaticus* (S. Joseph, unpub. data).

## Conclusions

The 7 housekeeping genes for MLST analysis are not virulence related, but a large proportion of severe neonatal infections were caused by a single sequence type. Whether this is caused by survival characteristics increasing persistence under desiccated conditions, and hence neonatal exposure or particular virulence capabilities, is uncertain. It is plausible that different age groups are exposed to different genotypes of *C. sakazakii* according to their diet and lifestyle. *C. sakazakii* ST4 appears to be a stable clone because strains have been isolated from 7 countries for >50 years. The earliest (1951) nonclinical isolate was from a can of dried milk (*13*). Whether this clonal nature occurs in other *Cronobacter* spp. awaits future investigation.
